# Pulmonary contusion in a collegiate diver: a case report

**DOI:** 10.1186/1752-1947-5-362

**Published:** 2011-08-10

**Authors:** Mathew W Lively

**Affiliations:** 1 Department of Internal Medicine, West Virginia University, P.O. Box 9167, Morgantown, WV 26506, USA

## Abstract

**Introduction:**

Pulmonary contusions typically occur after high-energy trauma and have rarely been reported as occurring during participation in sports. This is the first reported case of a pulmonary contusion occurring in a sport other than football.

**Case Presentation:**

A 19-year-old Caucasian man impacted the water awkwardly after diving off a one-meter springboard. He complained of chest discomfort and produced immediate hemoptysis. Computed tomography confirmed the diagnosis of pulmonary contusion. The athlete recovered without complications and returned to activity one week after injury.

**Conclusion:**

Immediate hemoptysis following blunt chest trauma during sports activity may indicate an underlying pulmonary contusion. No specific guidelines exist for return to athletic competition following pulmonary contusion, but a progressive return to activities once symptoms resolve appears to be a reasonable approach.

## Introduction

Chest and pulmonary injuries, including pneumothorax, pneumomediastinum, and subcutaneous emphysema, have been documented as a complication of sports activities [1,2]. Pulmonary contusions have rarely been reported as occurring during sports participation despite the fact that high-energy trauma is the most common mechanism for this injury. Immediate hemoptysis following trauma has also been rarely documented as an indicator of a possible pulmonary contusion. We describe the case of a diver who suffered a pulmonary contusion with immediate hemoptysis after his chest impacted the water.

## Case Presentation

An experienced 19-year-old Caucasian male collegiate diver was attempting a new dive from a one-meter springboard and hit the water awkwardly. The athlete impacted the water directly on his chest and commented that it felt as if he 'got hit by a truck.' He exited the pool complaining of mild anterior chest pain and within minutes began coughing associated with hemoptysis. He presented to the athletic training room where he continued to produce several milliliters of blood-tinged sputum for approximately twenty minutes. His mild chest pain persisted, but he denied dyspnea. His history was negative for any chronic medical disease, including pulmonary conditions, and he was not taking any medications.

**Figure 1:**
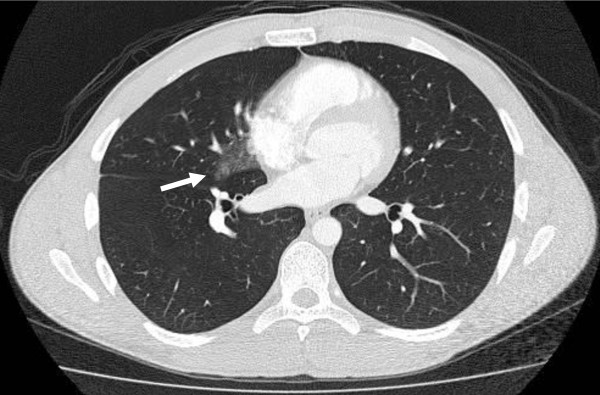
**Pulmonary contusion in the right lung**. CT of the chest obtained shortly after trauma showing opacity in the right middle lobe consistent with a pulmonary contusion.

The athlete was transported to the hospital emergency department where his vital signs and oxygen saturation were stable upon admission. The hemoptysis and chest pain had resolved and he complained only of mild chest pressure. Pulmonary auscultation revealed clear breath sounds with good air entry and he denied pain with deep inspiration. He had no tenderness to palpation over his chest or ribs and no subcutaneous crepitus was evident on his thorax or neck. A chest X-ray was not performed, but computed tomography (CT) of the chest revealed a ground-glass opacity in the medial portion of the right middle lobe consistent with a pulmonary contusion (Figure 1).

**Figure 2:**
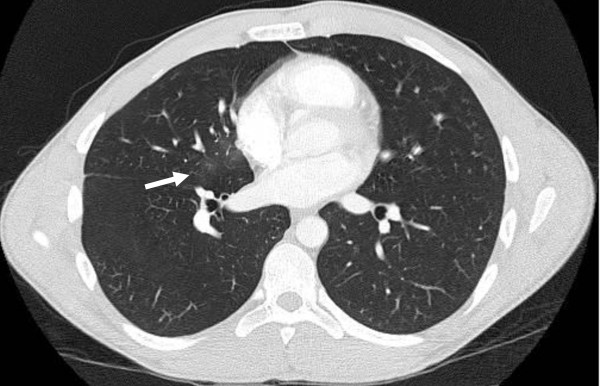
**Resolving pulmonary contusion**. CT of the chest one week after injury showing near-complete resolution of the pulmonary contusion.

He was withheld from diving activity for the next week and had no further episodes of hempotysis or chest pain. A repeat CT of the chest obtained seven days after the injury showed near complete resolution of the right middle lobe opacity (Figure 2). He was cleared to resume progressive activity and participated for the remainder of the season without sequelae.

## Discussion

Pulmonary contusion is the most common lung injury following blunt chest trauma [3,4] but has rarely been reported as a complication from participation in sports. Only three cases of pulmonary contusion in athletes have been documented in the literature with all of them occurring in football players [5,6]. The majority of pulmonary contusions are the result of high-energy blunt trauma, such as motor vehicle crashes, although there is a reported case of an individual suffering a contusion after being struck in the chest with a rubber bullet [7]. The athlete presented in this case was a diver whose only impact was with the water after jumping from a one-meter board.

Clinical manifestations of a pulmonary contusion often present acutely and may consist of dyspnea, tachypnea, chest pain, or hemoptysis [4,8]. Auscultation of the chest may reveal crackles or diminished breath sounds, but examination may be normal with small contusions [5]. Although the athlete in this case had a normal examination, his history of immediate hemoptysis following injury was consistent with the presentation of two football players in another case report [5].

Pulmonary contusions revealed on radiography appear as focal or diffuse infiltrations that may not conform to segments or lobes [3]. Conventional X-rays tend to underestimate the extent of lung damage and findings may not appear until four to six hours after injury and may take up to 48 hours to become evident [4,8,9]. CT is highly sensitive in detecting pulmonary contusions and the volume of lung involvement on CT scanning correlates with clinical outcomes [8-10]. Trauma patients with small pulmonary contusions (<18% of lung volume) have clinical outcomes that are equivalent to trauma patients without contusions [10].

Occult pulmonary contusions are defined as those evident on initial CT scanning but not seen on initial X-ray films. Recent studies investigating the clinical significance of such injuries have revealed that occult pulmonary contusions carry a better prognosis than contusions that appear on both studies [8-10]. In fact, these studies question the need for initial chest CT scanning in the detection of pulmonary contusions. Deunk *et al*. [10] showed that the presence of an occult pulmonary contusion did not influence the outcome of adult blunt trauma patients. Trauma patients with pulmonary contusions involving >18% of lung volume and those with contusions evident on initial chest X-ray had a higher morbidity and mortality than patients with smaller contusions or those with a normal X-ray [10]. Wylie *et al*. [9] reported the size of a pulmonary contusion determined by X-ray, but not CT, was correlated with the degree of abnormal gas exchange found in pediatric trauma patients. Since occult pulmonary contusions appear to have little clinical significance, initial chest X-rays seem to be of value in predicting the potential seriousness of a pulmonary contusion. CT scanning, however, is more accurate in determining the extent of lung involvement and is the most often used radiological study in blunt trauma patients [10]. The athlete in this case was evaluated in an emergency department where he received an initial chest CT and no conventional X-ray.

Treatment for pulmonary contusions is supportive. In general, respiratory derangements associated with pulmonary contusions often resolve within three to five days [4]. The most common complications are pneumonia and Adult Respiratory Distress Syndrome (ARDS), although these conditions typically occur only in patients with severe trauma and large diffuse pulmonary contusions [8,10]. Current data do not support the use of prophylactic antibiotics or corticosteroids in the treatment of pulmonary contusions [8].

We illustrate the case of a patient with pulmonary complication from diving activity, but the possibility of neck trauma, including cervical spine and carotid artery injuries, should also be considered when evaluating an athlete following diving-related trauma [11].

## Conclusions

Based on case reports, an athlete suffering blunt chest trauma followed by immediate hemoptysis should be suspected of having a pulmonary contusion. If initial chest X-rays are negative, recent evidence suggests an uncomplicated recovery is likely. There are no specific guidelines for return to athletic participation after pulmonary contusion, but in view of the benign nature of the reported injuries in this setting, a reasonable approach appears to be a progressive return to activity once symptoms resolve.

## Consent

Written informed consent was obtained from the patient for publication of this case report and any accompanying images. A copy of the written consent is available for review by the Editor-in-Chief of this journal.

## Competing interests

The author declares that they have no competing interests.
